# Coupled responses of thermomechanical waves in functionally graded viscoelastic nanobeams via thermoelastic heat conduction model including Atangana–Baleanu fractional derivative

**DOI:** 10.1038/s41598-024-58866-2

**Published:** 2024-04-20

**Authors:** Ahmed E. Abouelregal, Marin Marin, Abdelaziz Foul, S. S. Askar

**Affiliations:** 1 Department of Mathematics, Faculty of Science, Mansoura University, 35516 Mansoura, Egypt; 2 Department of Mathematics and Computer Science, Transilvania University of Brasov, 500036 Brasov, Romania; 3 Department of Statistics and Operations Research, College of Science, King Saud, Riyadh, 11451 Saudi Arabia; 4 Academy of Romanian Scientists, 050045, Bucharest, Romania

**Keywords:** Nonlocality, Viscoelastic nanobeams, AB fractional derivative, Heterogeneity, Materials science, Mathematics and computing, Nanoscience and technology

## Abstract

Accurately characterizing the thermomechanical parameters of nanoscale systems is essential for understanding their performance and building innovative nanoscale technologies due to their distinct behaviours. Fractional thermal transport models are commonly utilized to correctly depict the heat transfer that occurs in these nanoscale systems. The current study presents a novel mathematical thermoelastic model that incorporates a new fractional differential constitutive equation for heat conduction. This heat equation is useful for understanding the effects of thermal memory. An application of a fractional-time Atangana–Baleanu (AB) derivative with a local and non-singular kernel was utilized in the process of developing the mathematical model that was suggested. To deal with effects that depend on size, nonlocal constitutive relations are introduced. Furthermore, in order to take into consideration, the viscoelastic behaviour of the material at the nanoscale, the fractional Kelvin–Voigt model is utilized. The proposed model is highly effective in properly depicting the unusual thermal conductivity phenomena often found in nanoscale devices. The study also considered the mechanical deformation, temperature variations, and viscoelastic characteristics of the functionally graded (FG) nanostructured beams. The consideration was made that the material characteristics exhibit heterogeneity and continuous variation across the thickness of the beam as the nanobeam transitions from a ceramic composition in the lower region to a metallic composition in the upper region. The complicated thermomechanical features of simply supported viscoelastic nanobeams that were exposed to harmonic heat flow were determined by the application of the model that was constructed. Heterogeneity, nonlocality, and fractional operators are some of the important variables that contribute to its success, and this article provides a full study and illustration of the significance of these characteristics. The results that were obtained have the potential to play a significant role in pushing forward the design and development of tools, materials, and nanostructures that have viscoelastic mechanical characteristics and graded functions.

## Introduction

It's not possible to fully understand the elasticity of viscoelastic materials without looking at some basic properties and conducting extensive experiments to reveal their complex mechanical properties. By studying the elastic properties of viscoelastic materials, researchers and engineers can make and improve materials with specific viscoelastic properties. This makes it easier to use these materials in many fields, including materials science, biology, mechanical engineering, and the mechanics of structures^1^. Viscoelastic materials exhibit time-dependent deformation, indicating that the intensity and duration of the applied stress have an impact on their mechanical response. Creep, stress relaxation, and hysteresis comprise the time-dependent reaction. These show up as slow deformation under constant load, a decrease in stress with time under constant strain, and energy loss during the loading and unloading cycles^2^. Viscoelastic materials can also have frequency-dependent characteristics, wherein their mechanical behavior fluctuates in relation to the frequency of applied pressures or deformations. Dynamic mechanical analysis typically describes this behavior. This method measures the material's storage and loss moduli as frequency-dependent variables. In situations with dynamic loading or oscillating forces, it is very important to understand the frequency-dependent characteristics^3^.

The temperature dependence of the elastic behavior of viscoelastic materials can be significant. Temperature fluctuations can have a substantial impact on the viscoelastic properties of a material, leading to changes in its elastic modulus, damping characteristics, and overall mechanical performance. Considering temperature sensitivity is paramount when utilizing viscoelastic materials in various settings and under fluctuating thermal circumstances^4,5^. Creep refers to the progressive deformation of a substance when subjected to a sustained load, whereas stress relaxation denotes the steady decline in stress levels over some time when subjected to a continuous strain. Understanding viscoelastic processes is important for understanding how materials change shape and react to long-term mechanical stress, which in turn helps us figure out how stable and long-lasting they are^6^.

Fractional physical models are mathematics models that integrate fractional calculus operators to represent physical events. We also refer to these models as fractional-order models or non-integer-order models. Fractional physical models, on the other hand, require fractional derivatives or integrals, in contrast to conventional integer-order models, which are based on ordinary differential equations (ODEs) and involve integer deviations. The use of fractional calculus, which deals with derivatives and integrals of orders other than integers, allows for an accurate and flexible description of complex physical systems. It offers a method for describing systems that include memory effects, behaviour that is not local, and interactions that occur across considerable distances^7^. Numerous scientific and engineering disciplines, such as physics, chemistry, biology, engineering, and finance, have taken advantage of fractional physical models to solve their problems. The scenarios in which systems display non-local or non-Markovian behaviour, anomalous diffusion, power-law decay, or fractal properties are particularly advantageous for the use of these techniques. In the field of fractional calculus, several significant fractional derivative operators are frequently utilised to describe and analyse physical systems. The Riemann–Liouville fractional derivative is one of the first expressions of fractional derivatives, and it is also one of the most commonly used expressions^8^. Additionally, the Grunwald–Letnikov fractional derivative is an example of a discrete approximation of fractional derivatives. Among the fractional derivatives that have non-anomalous and non-local kernels, two examples include the Atangana and Baleanu^9^ and Caputo and Fabrizio^10^ fractional derivative operators. To describe the dynamics and characteristics of complex systems, these operators have been utilised in a variety of domains, including physics, engineering, and mathematical modeling.

Functionally gradient materials (FGMs) are a type of engineering material that is designed and manufactured to exhibit a continuous change in composition, microstructure, and properties along a specific spatial dimension. The progressive variation of material qualities observed in FGMs enables the incorporation of diverse materials, each selected for its own functionality. Consequently, FGMs exhibit superior overall performance compared to homogeneous materials traditionally employed^11^. FGMs have been widely utilized in diverse industries such as aerospace engineering, automobile manufacturing, medical technology, thermal management systems, and sophisticated equipment for structural components. Illustrative instances of applications encompass the advancement of heat-resistant components designed for gas turbines, medical devices exhibiting enhanced osseointegration, and high-performance material structures tailored for space exploration endeavors^12^. The FGM materials can be intentionally designed to exhibit certain thermal expansion parameters, enabling them to effectively endure temperature gradients and minimize the likelihood of thermal stresses and related failures. This attribute is especially beneficial in situations with elevated temperatures and thermal shock circumstances^13^. By mixing different materials with properties that complement each other, FGM can be made to have specific properties, such as better resistance to wear, thermal insulation, electrical conductivity, or biocompatibility. The ability to customize materials allows for the creation of innovative materials that can be utilized in a wide range of applications across various sectors^14^. The ongoing investigation and progress in the field of functionally graded materials persistently broaden their prospective applications and contribute to the progression of materials science and engineering. This, in turn, offers inventive resolutions for tackling intricate engineering dilemmas and enhancing the efficiency and dependability of cutting-edge technological systems. Various articles have discussed the static and dynamic characteristics of functionally graded material and its use in engineering construction^15–19^.

Nanoelectromechanical systems, often known as NEMS, are a type of nanoscale device that integrates electrical and mechanical capabilities. These systems work at the nanoscale. Microelectromechanical systems (MEMS) serve as the basis for the development of NEMS technology, which, in addition to offering enhanced performance characteristics, also makes it possible to further miniaturize components. To obtain expanded functionality and capabilities, NEMS devices make use of the one-of-a-kind qualities that are displayed by materials at the nanoscale. Quantum effects and surface forces become more evident at such small dimensions, which enables completely new device behavior and performance to be realized^20,21^. Nanoscale nanoelectromechanical systems (NEMS) devices display unprecedented degrees of miniaturization, which enables the inclusion of complex functions into a single minuscule piece of equipment. The attribute above is of utmost importance in developing sophisticated sensing, actuation, and communication systems that necessitate compact and energy-efficient elements^22^. NEMS devices at the nanoscale are commonly characterized by their low-power operation, rendering them very energy-efficient and well-suited for integration into portable electronic devices, Internet of Things devices, and various other applications reliant on battery power. The advantageous characteristic of low power consumption is particularly significant in applications like remote sensing and monitoring, where there may be limitations on available power resources^23^. Although nanoscale NEMS devices provide some benefits, they also pose obstacles concerning their manufacture, integration, and dependability. Researchers and engineers persist in exploring novel manufacturing processes, materials, and design approaches to tackle these problems and unleash the full capabilities of nanoscale NEMS technology. These endeavors have resulted in notable progress in developing durable and dependable nanoscale NEMS devices for various applications, such as bioelectronics, nanorobotics, and quantum computing, among others.

Nonlocal elasticity theories have a crucial impact on the examination and development of sophisticated materials, structures, and devices, particularly when dealing with tiny length scales where conventional continuum mechanics theories may not be suitable. These theoretical frameworks offer a more profound comprehension of the complicated mechanical phenomena exhibited by materials possessing intricate microstructures and interfaces^24^. This, in turn, aids in advancing novel materials and structures that exhibit improved performance and dependability.

Two significant factors that alter the mechanical characteristics of nanobeams are the small-scale effect and the surface energy effect^25^. Previous research has investigated these phenomena through the utilization of experiments conducted at the nanoscale and in atomistic models. Moreover, the investigations above have provided evidence indicating that classical continuum mechanics models cannot capture any of these impacts. Consequently, researchers have proposed numerous non-classical continuum models over time to effectively represent the size-dependent characteristics of small structures. These models include the strain gradient Mindlin's elasticity theory^26^, the strain-driven Eringen's nonlocal model^27,28^, the stress-driven Romano and Barretta's model^29^, and other coupled concepts such as the nonlocal strain gradient theory constructed by Lim^30^. Barretta and Marotti de Sciarra^29^ also introduced the two-phase local/nonlocal stress and strain gradient model. Romano et al.^31^ have extensively debated the potential for mathematical errors in Eringen's model. The primary cause of this is the inherent mismatch between the equilibrium requirements and the higher-order constitutive boundary conditions.

Thermoelasticity is a viable approach for analyzing materials and structures subjected to modest temperature gradients, and it finds extensive use across many engineering disciplines. Usually, thermoelasticity is based on the Fourier heat conduction equation and the idea that temperatures should be equal in a certain area^32^. This theory explains the interaction between temperature gradient and mechanical deformation within materials, taking into account the effect of temperature changes on material hardness and thermal expansion^33^. Generalized thermoelasticity models^34–43^ encompass the fundamental principles of conventional thermoelasticity while incorporating additional considerations, such as the limited wave speeds of thermal disturbances and the non-instantaneous response of materials to temperature variations. These theories look at how the thermal and mechanical fields interact with each other. This helps us get a better picture of the transient thermoelastic phenomena that happen in materials when the temperature changes quickly or when they are loaded and unloaded quickly.

Moore–Gibson–Thompson (MGT) thermoelasticity theory is a new theoretical framework that extends traditional thermoelasticity theory by including the effect of the concept of thermal relaxation times on the transmission of thermal waves within elastic materials. Quintanilla^44,45^ formulated the hypothesis above with the aim of offering a more all-encompassing depiction of the transitory thermal and mechanical phenomena seen in materials when exposed to abrupt alterations in temperature. In recent years, the number of studies focusing on developing the theory of thermoelasticity (MGT) has witnessed significant growth^46–50^. Later studies have changed the extended thermoelastic theory into fractional ones by adding different time-fractional derivatives to hyperbolic heat transfer and mass diffusion equations^51,52^. The growing number of fractional calculus applications in both science and engineering served as the impetus for this extension. Due to the vast availability of energy sources that can be readily collected, the micro- and nanoscale vibration-based piezoelectric energy harvester has rapidly become an essential branch of the major emphasis area in modern times. Regarding piezoelectric nanostructures, thermoelastic diffusion, viscoelastic composite structures, and time-fractional order strain, references^53–59^ also provided a comprehensive assessment of current accomplishments and fundamental formulations in the field.

The development of the nonlocal thermo-viscoelastic fractional order model for functionally graded nanoscale beams is a notable progress in the realm of advanced materials and nano-mechanics. This model offers researchers and engineers a potent tool to explore and create functionally graded materials with tailored features. Consequently, it aids in creating cutting-edge and durable nanoscale gadgets and structures for diverse engineering purposes.

The main goal of this work is to study the vibrational behavior of functionally graded (FG) nanoscale viscoelastic beams under a variety of different environmental conditions. We used the theory of non-local elasticity to capture non-local effects on a small scale, and we considered fractional calculus to calculate memory effects. We also used the Kelvin–Voigt fractional viscoelastic model to describe how nanobeams behave in terms of viscoelastic properties. This model is based on the Atangana and Baleanu (AB) differential operators. The Bernoulli–Euler beam theory, a classical beam theory ideal for thin beams that experience modest deformations, was also considered to model the nanobeams investigated in this work. The non-local Bernoulli–Euler beam theory, the fractional Kelvin–Voigt viscoelastic model, and the theory of thermoelasticity are all combined in this study. This gives a complete picture of how FG nanoscale viscoelastic beams behave when they are heated and stressed. Taking into account the effects of small-scale, non-local, and relaxation time, the study explores the effect of different final conditions on the vibrational response of the beam. Finally, the governing equations incorporate a new fractional-order time derivative with a non-singular and non-local kernel. This inclusion allows a more precise depiction of the complex dynamics exhibited by the FG nanopackage as well as its memory-dependent properties.

The proposed model is used to analyze the thermomechanical interactions of nanobeams made of functionally graded (FG) thermo-viscoelastic material that are simply supported and exposed to harmonic thermal flux. The equation for transverse vibration is obtained by the use of Euler–Bernoulli beam theory and Hamilton's principle. A system of governing equations was established and solved utilizing the Laplace transform method in accordance with the given issue. Numerical findings are provided to analyze how the fractional order parameter, nonlocal parameter, and power law index affect the physical variables in the system. The findings from this study may be used to identify and describe different nanostructures, such as nano-electromechanical systems (NEMS), nano-actuators, and others. Studying how functionally graded nano-scale viscoelastic beams vibrate offers important information for designing, optimizing, and analyzing the performance of these nano-structures in many engineering applications.

## Formulation

### Fractional Kelvin–Voigt model

In the context of homogeneous and isotropic substances, the constitutive equations and strain–displacement relations can be formulated as follows^43^:

1 $${\tau }_{ij}=2\mu {e}_{ij}+\lambda {e}_{kk}{\delta }_{ij}-\gamma \theta {\delta }_{ij},$$

2 $$2{e}_{ij}={u}_{j,i}+{u}_{i,j},$$

where $\lambda$ and $\mu$ are the Lame's constants, ${\delta }_{ij}$ is the Kronecker delta, ${\tau }_{ij}$ are stress components, ${e}_{ij}$ are strain components, ${u}_{i}$ are displacement vector’s component, $\gamma =\left(3\lambda +2\mu \right){\alpha }_{t}$ is the thermal elastic coupling, ${\alpha }_{t}$ represents the coefficient of linear thermal expansion, $\theta =T-{T}_{0}$ is the temperature variation, the variable $T$ represents the absolute temperature, and ${T}_{0}$ denotes the temperature of the medium in its inherent condition. The Young's modulus ($E$) and the Poisson's ratio ($\nu$) are connected to the Lamé parameters, $\lambda$ and $\mu$, in the following ways:

3 $$\lambda =\frac{E\nu }{\left(1+\nu \right)\left(1-2\nu \right)}, \mu =\frac{E}{2\left(1+\nu \right)}.$$

The equation of motion governs the dynamic response of a thermoelastic body to external forces $\left({F}_{i}\right)$ and temperature variations. The equation of motion for a thermoelastic body can be expressed under the assumptions of isotropy and homogeneity, taking into account that the deformations are of relatively small magnitude, as follows^43^:

4 $${\sigma }_{ji,j}+{F}_{i}=\rho \frac{{\partial }^{2}{u}_{i}}{\partial {t}^{2}}.$$

The classical Kelvin–Voigt model, a widely recognized viscoelastic model, is employed to characterize the mechanical response of materials that have combined viscous and elastic characteristics. In order to take into account a substance's viscoelastic properties, especially within the Kelvin–Voigt model, the traditional Young's modulus ($E$) is changed to include the effect of viscosity. This model is commonly used to represent the viscoelastic behaviour of materials, where both the rate of deformation influences their mechanical properties over time and the duration of applied loads. The modified Young's modulus is expressed as^60^:

5 $$E={E}_{0}\left(1+{\tau }_{{\text{v}}}\frac{\partial }{\partial t}\right),$$

where ${E}_{0}$ is the elastic Young's modulus and ${\tau }_{{\text{v}}}$ is the vicosity or viscous damping coefficient. Substituting Eqs. (3) and (5) into Eq. (1) leads to^61^:

6 $${\tau }_{ij}={E}_{0}\left(1+{\tau }_{{\text{v}}}\frac{\partial }{\partial t}\right)\left[{\frac{1}{\left(1+\nu \right)}e}_{ij}+\frac{\nu }{\left(1+\nu \right)\left(1-2\nu \right)}{e}_{kk}{\delta }_{ij}-\frac{{\alpha }_{t}}{\left(1-2\nu \right)}\theta {\delta }_{ij}\right].$$

The fractional Kelvin–Voigt model is an expansion of the conventional Kelvin–Voigt model, widely employed for characterizing the viscoelastic properties of materials. A fractional Kelvin–Voigt viscoelastic beam in this study displays viscoelastic properties and is characterised by the fractional Kelvin–Voigt model. Adding fractional calculus to the Kelvin–Voigt model makes it better at showing how viscoelastic materials are, especially when the material behaves in complicated ways, like when it has traits that are passed down through generations or when it remembers things over time.

The constitutive equation governing the behavior of the fractional Kelvin–Voigt model can be expressed as follows^62^:

7 $${\tau }_{ij}={E}_{0}\left(1+{\tau }_{{\text{v}}}^{\alpha }{D}_{t}^{\alpha }\right)\left[{\frac{1}{\left(1+\nu \right)}e}_{ij}+\frac{\nu }{\left(1+\nu \right)\left(1-2\nu \right)}{e}_{kk}{\delta }_{ij}-\frac{{\alpha }_{t}}{\left(1-2\nu \right)}\theta {\delta }_{ij}\right].$$

In Eq. (7), ${D}_{t}^{\alpha }$ is fractional differential operator of order $\alpha$, ($0<\alpha <1$). Among the many definitions of fractional derivatives, the definition is often used in the Riemann–Liouville sense, which is defined using the relation^7,8^:

8 $${D}_{t}^{\alpha }\mathcal{f}\left(t\right)=\frac{1}{\Gamma \left(1-\alpha \right)}\frac{d}{dt}\underset{0}{\overset{t}{\int }}\frac{\mathcal{f}(\mathcal{s})}{{\left(t-\mathcal{s}\right)}^{\alpha }}d\mathcal{s}, 0<\alpha <1,$$

where $\Gamma \left(1-\alpha \right)$ denotes the Gamma function.

Recently, other formulations of fractional derivatives involving non-singular kernels have been proposed, such as the Caputo and Fabrizio^10^, Atangana and Baleanu^9^ fractional operators. Standard fractional derivatives can be used with these fractional derivative operators, which allows systems that have genetic and memory effects to be represented and studied. The importance of fractional derivatives with non-singular nuclei derives from the observation that some models of dissipative events cannot be adequately described by conventional fractional operators.

In the present work, the Atangana and Baleanu^9^ fractional derivative operators will be considered, which is defined as follows:

9 $${D}_{t}^{\alpha }\mathcal{f}\left(t\right)=\frac{1}{\left(1-\alpha \right)}\underset{0}{\overset{t}{\int }}{E}_{\alpha }\left[-{\mu }_{\alpha }{\left(t-\mathcal{s}\right)}^{\alpha }\right]\frac{d\mathcal{f}(\mathcal{s})}{d\mathcal{s}}d\mathcal{s}, \alpha \in \left(\mathrm{0,1}\right),$$

where ${\mu }_{\alpha }=\frac{\alpha }{\left(1-\alpha \right)}$ and ${E}_{\alpha }$ is the Mittag–Leffler function.

### Nonlocal theory of elasticity

The nonlocal integral theory of elasticity looks at the stress field at a certain point in space by combining the elastic strain field with the right averaging kernel and doing an integral convolution. When applied to fields that change slowly over a characteristic distance, Eringen^28,63^ showed that integral-type constitutive equations can be boiled down to partial differential equations. The Eringen model^28^ states that the differential constitutive relation can be used to describe the nonlocal constitutive behaviour of a Hookean solid as follows:

10 $$\left(1-\xi {\nabla }^{2}\right){\sigma }_{ij}={\tau }_{ij},$$

where ${\sigma }_{ij}$ is the nonlocal stress tensor, ${\nabla }^{2}$ is Laplacian operator and $\xi ={\left({e}_{0}a\right)}^{2}$ is the nonlocal parameter in which ${e}_{0}$ represents the constant of the material and ${e}_{0}$ represents the internal characteristic lengths.

### Moore–Gibson–Thompson thermoelastic theory

In the context of classical thermoelasticity, the fundamental set of field equations encompasses the classic Fourier law of heat conduction. When these equations are simplified to the displacement-temperature field equations, they exhibit characteristics of a hyperbolic-parabolic nature. This means that a traditional thermoelastic body's response to thermomechanical stress has a propagation velocity that is not limited. Since 1967, a collection of theories known as generalised thermoelasticity theories have been formulated^34–40^. The models proposed by Green and Naghdi^37,38^ have garnered significant interest in the field due to their contributions to generating three distinct forms of generalised thermoelasticity theories. This has subsequently motivated scholars to pursue further investigations in this particular area.

Fourier's law has been redefined according to the GN-III model as^37^

11 $${q}_{i}=-K{\theta }_{,i}-{K}^{*}{\mathcal{V}}_{,i}.$$

In this context, the symbol $K$ is used to represent the thermal conductivity of the material, ${K}^{*}$ is the rate of conductivity, $q$ is used to represent the heat flux vector and $\mathcal{V}$ symbolizes the thermal displacement in which $\dot{\mathcal{V}}=\theta$.

The GN-III model similarly predicts the unbounded propagation of thermal waves, consistent with Fourier's conventional law^64^. Hence, the introduction of a modified version of Eq. (11) has been proposed in order to address this seeming challenge. In a recent study, Quintanilla^44,45^ introduced a novel theory of thermoelasticity in which the Moore–Gibson–Thompson (MGT) equation serves to describe the heat conduction equation. The MGT thermoelasticity concept is a novel theoretical framework that extends the concepts of the LS theory and the GN-III model in the field of thermoelasticity. The incorporation of a relaxation factor in Eq. (11) has been implemented in this revision, resulting in^44^

12 $${\left(1+{\tau }_{0}\frac{\partial }{\partial t}\right)q}_{i}=-K{\theta }_{,i}-{K}^{*}{\mathcal{V}}_{,i}.$$

The symbol ${\tau }_{0}$ represents the parameter associated with time relaxation. Numerous studies (see for example^65^) have obtained experimental values for ${\tau }_{0}$.

The entropy heat equation is a mathematical formulation that characterises the transfer of thermal energy inside a system by using the concept of entropy. A basic understanding of the relationship between entropy and heat transport is provided by this idea, which is often used to look at irreversible thermodynamic phenomena. The entropy thermal equation is constructed from the second rule of thermodynamics, which postulates that the entropy of an isolated system remains constant or increases with time. The entropy-heat (energy) equation can be expressed as follows:

13 $${-q}_{i,i}=\rho {C}_{E}\frac{\partial \theta }{\partial t}+\frac{{\alpha }_{t}{T}_{0}{E}_{0}}{\left(1-2\nu \right)}\left(1+{\tau }_{{\text{v}}}^{\alpha }{D}_{t}^{\alpha }\right)\frac{\partial {e}_{kk}}{\partial t}-Q.$$

By combining Eqs. (12) and (13), we may obtain the MGT thermo-viscoelastic heat transfer model with fractional order derivatives as follows

14 $${\left(K\frac{\partial {\theta }_{,i}}{\partial t}+{K}^{*}{\theta }_{,i}\right)}_{,i}=\left(1+{\tau }_{0}\frac{\partial }{\partial t}\right)\left[\rho {C}_{E}\frac{{\partial }^{2}\theta }{\partial {t}^{2}}+\frac{{\alpha }_{t}{T}_{0}{E}_{0}}{\left(1-2\nu \right)}\left(1+{\tau }_{{\text{v}}}^{\alpha }{D}_{t}^{\alpha }\right)\frac{{\partial }^{2}{e}_{kk}}{\partial {t}^{2}}-\frac{\partial Q}{\partial t}\right].$$

Pellicer and Quintanilla^66^ have demonstrated the uniqueness and instability of solutions in the concept of thermoelasticity, specifically in relation to the MGT equation. In a recent study conducted by Bazarra et al.^67^, the numerical aspects of the MGT thermoelastic theory were examined. The discrete stability property was also demonstrated in this framework.

## Formulation of the problem

**Figure 1:**
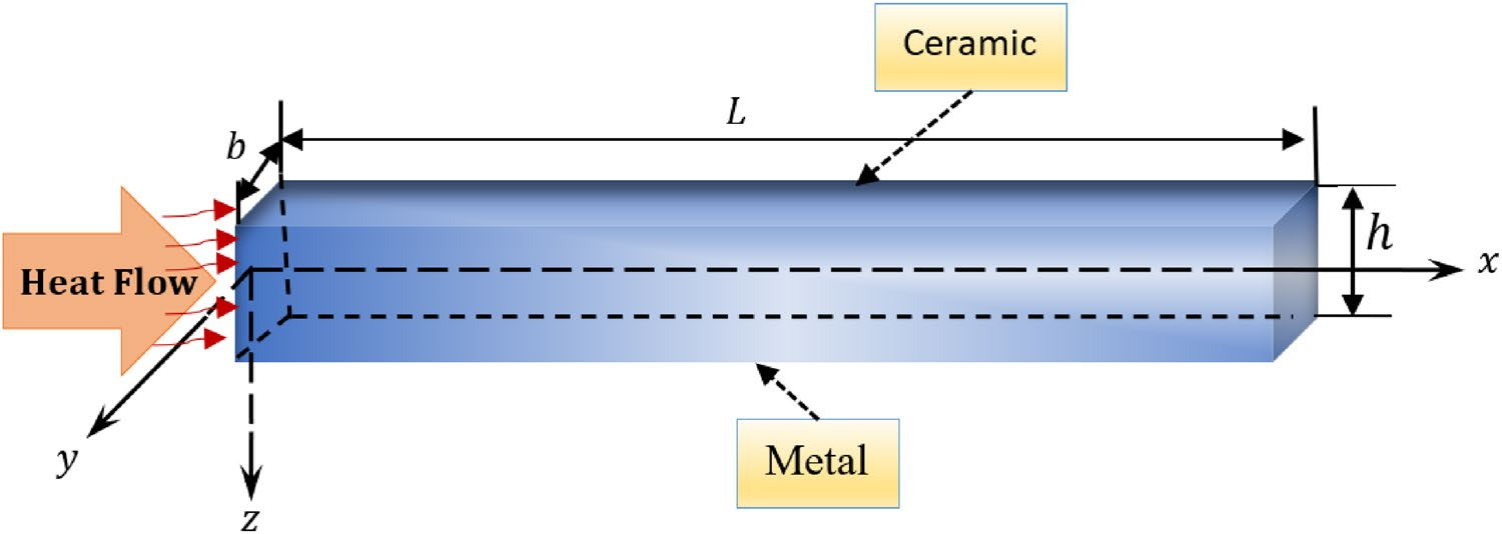
A schematic of thermo-viscoelastic FG nanobeam.

A thermoelastic solid nanobeam with functionally graded properties in the Cartesian coordinate system $Oxyz$ will be considered in this study. The $x$-axis is aligned with the axial direction of the beam, while the $y$ and $z$ axes represent the breadth and thickness, respectively (Fig. 1). At the state of equilibrium, the FG beam remains in a condition of zero strain, stress, and uniform temperature ${T}_{0}$ across its entirety. The analysis focuses on the small flexural deflections exhibited by a nanobeam, which has dimensions of length $L$ (where $0\le x\le L$), width $b$ (where $-b/2\le x\le b/2$), and thickness $h$ (where $-h/2\le x\le h/2$. The basic equations governing motion and heat transfer will be considered in the context of generalized (non-Fourier) MGT thermoelasticity. The case will be taken into account in which there are neither body forces, nor external loads (${F}_{i}=0$) nor even external heat sources ($Q=0$).

There is a smooth change in the material properties only in the $z$-direction thickness of the nanobeam, which is made of an elastic and isotropic functionally graded material. The FG nanobeam's material properties, such as its Young's modulus ($E$), thermal conductivity ($K$), thermal expansion (${\alpha }_{t}$), and mass density ($\rho$), are thought to change continuously along the thickness direction ($z$-axis direction). The power function $P\left(z\right)$ models this variation by relating the values to the volume fractions of the constituents.

Material properties over thickness show variations depending on the volume fraction, which can be expressed as

15 $$P\left(z\right)={P}_{m}{e}^{{n}_{p}(2z-h)/h}, {n}_{p}=ln\sqrt{{P}_{m}/{P}_{c}} .$$

Here, ${n}_{p}$ stands for the gradient index, which determines the progressive change in material properties that occurs through the thickness of the nanobeam. The variable ${P}_{c}$ represents the material property of the pure ceramic, while ${P}_{m}$ represents the material property of the pure metal. It is important to acknowledge that the material properties of the beam under consideration exhibit a metal-rich composition at the lower surface, located at $z=h/2$, and a ceramic-rich composition at the upper surface, located at $z=-h/2$. When the value of ${n}_{p}$ is set as zero, the beam will exhibit homogeneity, indicating that the material composition will consist only of ceramic.

The equations of motion are determined using the principles of Euler–Bernoulli beam theory. Based on this theory, the displacement field at any position along the beam can be expressed as follows:

16 $$u\left(x,z,t\right)=-z\frac{\partial w}{\partial x},\mathrm{ v}=0, w\left(x,y,z,t\right)=w\left(x,t\right),$$

where $w$ is the deflection.

In the context of fractional modeling and viscoelastic functionally graded materials (FGMs), the Young's modulus ${\widetilde{E}}_{m}$ of the material can be represented mathematically as follows:

17 $${\widetilde{E}}_{m}={E}_{m}\left(1+{\tau }_{{\text{v}}}^{\alpha }{D}_{t}^{\alpha }\right).$$

In this representation, the utilisation of fractional order modelling makes it possible to provide a more precise description of the behaviour of the viscoelastic material. Additionally, it provides us with a means to examine and speculate on the behaviour of FGM viscoelastic beams when subjected to many distinct stresses. Using Eqs. (15)–(17), the MGT heat transfer Eq. (14) for the nanobeam can be derived as follows

18 $$\begin{aligned} & \left( {1 + \tau_{0} \frac{\partial }{\partial t}} \right)\left[ {\rho_{m} C_{Em} e^{{n_{{\rho C_{E} }} H\left( z \right)}} \frac{{\partial^{2} \theta }}{{\partial t^{2} }} - {\text{z}}\gamma_{m} e^{{n_{\gamma } H\left( z \right)}} T_{0} \left( {1 + \tau_{{\text{v}}}^{\alpha } D_{t}^{\alpha } } \right)\frac{{\partial^{4} w}}{{\partial t^{2} \partial x^{2} }}} \right] \\ & \quad = \left[ {K_{m} e^{{n_{K} H\left( z \right)}} \frac{\partial }{\partial t} + K_{m}^{*} e^{{n_{{K^{*} }} H\left( z \right)}} } \right]\left[ {\frac{{\partial^{2} \theta }}{{\partial x^{2} }} + \frac{{\partial^{2} \theta }}{{\partial z^{2} }} + \frac{{2n_{K} }}{h}\frac{\partial \theta }{{\partial z}}} \right], \\ \end{aligned}$$

where

19 $$H\left(z\right)=\frac{2z-h}{h},{\gamma }_{m}=\frac{{E}_{m}{\alpha }_{m}}{1-2{\nu }_{m}}, {\rho }_{m}{C}_{Em}=\frac{{K}_{m}}{{\chi }_{m}}.$$

In accordance with Eq. (18), it is important to take notice that the coefficients ${n}_{K}$, ${n}_{\gamma }$, ${n}_{{K}^{*}}$, and ${n}_{\rho {C}_{E}}$ are established by considering the characteristics of ceramic and metal materials.

Assuming that there is no transfer of thermal energy between the upper and lower edges of the nanobeam under study, and that its upper and lower edges are effectively thermally insulated. It has been suggested that there is no temperature difference between the top and bottom surfaces of the nanobeam, and as a result, $\frac{\partial \theta }{\partial z}=0$ at $z=\pm h/2$. It will also be taken into account that the temperature changes in a sinusoidal form along the thickness direction ($z$ direction) of the nanobeam, and this means that the temperature distribution as a function of $z$ can be expressed in the form of a sine function as follows:

20 $$\theta \left(x,z,t\right)=\Theta \left(x,t\right){\text{sin}}\left(\frac{\pi z}{h}\right).$$

By substituting Eq. (20) into Eq. (18) and performing integration throughout the nanobeam thickness from $-h/2$ to $h/2$ with respect to $z$, the following equation can be obtained

21 $$\left(\frac{\partial }{\partial t}+\frac{{K}_{m}^{*}}{{K}_{m}}\right)\frac{{\partial }^{2}\Theta }{\partial {x}^{2}}=\left(1+{\tau }_{0}\frac{\partial }{\partial t}\right)\left[{\overline{\mu }}_{\rho {C}_{E}}\eta \frac{{\partial }^{2}\Theta }{\partial {t}^{2}}-\frac{{{\overline{\mu }}_{\gamma }\gamma }_{m}h{T}_{0}}{{K}_{m}}\left(1+{\tau }_{{\text{v}}}^{\alpha }{D}_{t}^{\alpha }\right)\frac{{\partial }^{4}w}{\partial {t}^{2}\partial {x}^{2}}\right],$$

where

22 $$\begin{aligned} \mu_{{\rho C_{E} }} = & \frac{{2n_{{\rho C_{E} }} \left( {1 + e^{{ - 2n_{{\rho C_{E} }} }} } \right)}}{{\pi^{2} + 4\left( {n_{{\rho C_{E} }} } \right)^{2} }}, \mu_{K} = \frac{{2n_{K} \left( {1 + e^{{ - 2n_{K} }} } \right)}}{{\pi^{2} + 4\left( {n_{K} } \right)^{2} }},\overline{\mu }_{\gamma } = \frac{{\mu_{\gamma } }}{{\mu_{K} }}, \\ \mu_{\gamma } = & \frac{{n_{\gamma } \left( {1 + e^{{ - 2n_{\gamma } }} } \right) + e^{{ - 2n_{\gamma } }} - 1}}{{4\left( {n_{\gamma } } \right)^{2} }},\eta = \frac{{\rho_{m} C_{Em} }}{{K_{m} }},\overline{\mu }_{{\rho C_{E} }} = \frac{{\mu_{{\rho C_{E} }} }}{{\mu_{K} }}. \\ \end{aligned}$$

Equations (15) and (16) can be used to obtain the foundational equation that includes the dimensionless nonlocal axial stress ${\sigma }_{xx}$, which appears in Eq. (10), based on the nonlocal theory of thermoviscoelasticity as follows:

23 $${\sigma }_{xx}-\xi \frac{{\partial }^{2}{\sigma }_{xx}}{\partial {x}^{2}}={-E}_{m}\left(1+{\tau }_{{\text{v}}}^{\alpha }{D}_{t}^{\alpha }\right){e}^{{n}_{E\alpha }H\left(z\right)}\left[z\frac{{\partial }^{2}w}{\partial {x}^{2}}+{\alpha }_{m}\theta \right],$$

where $${n}_{E\alpha }={\text{ln}}\sqrt{{E}_{m}{\alpha }_{m}/{E}_{c}{\alpha }_{c}}$$.

The equation for the bending moment, denoted as $M$, can be derived using Eq. (23) as

24 $$M-\xi \frac{{\partial }^{2}M}{\partial {x}^{2}}={-b{h}^{2}E}_{m}\left(1+{\tau }_{{\text{v}}}^{\alpha }{D}_{t}^{\alpha }\right)\left[{h\mu }_{E}\frac{{\partial }^{2}w}{\partial {x}^{2}}+{\alpha }_{m}{\mu }_{E\alpha }\Theta \right],$$

in which

25 $$\begin{aligned} \mu_{K} = & \frac{{2n_{E\alpha } \left( {\pi^{2} + 4n_{E\alpha }^{2} } \right)\left( {1 - e^{{ - 2n_{E\alpha } }} } \right) + \left( {\pi^{2} - 4n_{E\alpha }^{2} } \right)\left( {1 + e^{{ - 2n_{E\alpha } }} } \right)}}{{\left( {\pi^{2} + 4n_{E\alpha }^{2} } \right)^{2} }}, \\ \mu_{E} = & \frac{{\left( {n_{E}^{2} + 2} \right)\left( {1 - e^{{ - 2n_{E} }} } \right) - 2n_{E} \left( {1 - e^{{ - 2n_{E} }} } \right)}}{{8n_{E}^{3} }}. \\ \end{aligned}$$

The application of Hamilton's principle to micro/nano beams results in the derivation of the Euler–Bernoulli beam transverse motion equation, which is utilized to characterize the lateral displacement of the beam. Based on the expanded Hamiltonian principle, the following equation of motion can be obtained:

26 $$\frac{{\partial }^{2}M}{\partial {x}^{2}}=\frac{\left(1-{e}^{{-2n}_{\rho }}\right){\rho }_{m}}{{2n}_{\rho }}A\frac{{\partial }^{2}w}{\partial {t}^{2}}.$$

When Eq. (24) is combined into Eq. (26), the beam motion equation results as follows:

27 $$\left(1+{\tau }_{{\text{v}}}^{\alpha }{D}_{t}^{\alpha }\right)\left[\frac{{\partial }^{4}w}{\partial {x}^{4}}+\frac{{\alpha }_{m{\mu }_{E\alpha }}}{{\mu }_{E}}\frac{{\partial }^{2}\Theta }{\partial {x}^{2}}\right]+\frac{{\rho }_{m}\left(1-{e}^{{-2n}_{\rho }}\right)}{{2{E}_{m}{h}^{2}n}_{\rho }{\mu }_{E}}\left(\frac{{\partial }^{2}w}{\partial {t}^{2}}-\xi \frac{{\partial }^{4}w}{\partial {t}^{2}\partial {x}^{2}}\right)=0.$$

The bending moment given in Eq. (24) can be represented as follows when applying expression (26):

28 $$M\left(x,t\right)=\xi A\frac{\left(1-{e}^{{-2n}_{\rho }}\right){\rho }_{m}}{{2n}_{\rho }}\frac{{\partial }^{2}w}{\partial {t}^{2}}{-b{h}^{2}E}_{m}\left(1+{\tau }_{{\text{v}}}^{\alpha }{D}_{t}^{\alpha }\right)\left[{h\mu }_{E}\frac{{\partial }^{2}w}{\partial {x}^{2}}+{\alpha }_{m}{\mu }_{E\alpha }\Theta \right].$$

In order to facilitate analysis and calculation, the following variables are presented dimensionless:

29 $$\begin{array}{c}\left\{{x}^{\mathrm{^{\prime}}},{z}^{\mathrm{^{\prime}}},{u}^{\mathrm{^{\prime}}},{w}^{\mathrm{^{\prime}}},{L}^{\mathrm{^{\prime}}},{h}^{\mathrm{^{\prime}}}\right\}={{c}_{0}\eta }_{0}\left\{x,z,u,w,L,h\right\}, \left\{{t}^{\mathrm{^{\prime}}},{\tau {\prime}}_{0},{\xi }^{\mathrm{^{\prime}}}\right\}={c}_{0}^{2}{\eta }_{0}\left\{t,{\tau }_{0},{\eta }_{0}\xi \right\}, {\Theta }^{\mathrm{^{\prime}}}=\frac{\Theta }{{T}_{0}}\end{array}.$$

After using the nondimensional variables (29) in Eqs. (21), (27), and (28) and removing the prime, it is possible to obtain:

30 $$\left(1+{\tau }_{{\text{v}}}^{\alpha }{D}_{t}^{\alpha }\right)\frac{{\partial }^{4}w}{\partial {x}^{4}}+{A}_{1}\left(\frac{{\partial }^{2}w}{\partial {t}^{2}}-\xi \frac{{\partial }^{4}w}{\partial {t}^{2}\partial {x}^{2}}\right)=-{A}_{2}\left(1+{\tau }_{{\text{v}}}^{\alpha }{D}_{t}^{\alpha }\right)\frac{{\partial }^{2}\Theta }{\partial {x}^{2}},$$

31 $$\left(\frac{\partial }{\partial t}+{\omega }_{0}\right)\frac{{\partial }^{2}\Theta }{\partial {x}^{2}}=\left(1+{\tau }_{0}\frac{\partial }{\partial t}\right)\left[{A}_{3}\frac{{\partial }^{2}\Theta }{\partial {t}^{2}}-{A}_{4}\left(1+{\tau }_{{\text{v}}}^{\alpha }{D}_{t}^{\alpha }\right)\left(\frac{{\partial }^{4}w}{\partial {t}^{2}\partial {x}^{2}}\right)\right],$$

32 $$M\left(x,t\right)={A}_{1}\left(\xi \frac{{\partial }^{2}w}{\partial {t}^{2}}-\left(1+{\tau }_{{\text{v}}}^{\alpha }{D}_{t}^{\alpha }\right)\frac{{\partial }^{2}w}{\partial {x}^{2}}\right)-{A}_{2}\left(1+{\tau }_{{\text{v}}}^{\alpha }{D}_{t}^{\alpha }\right)\Theta ,$$

where

33 $${A}_{1}=\frac{\left(1-{e}^{{-2n}_{\rho }}\right)}{{2{h}^{2}n}_{\rho }{\mu }_{E}},{A}_{2}=\frac{{{T}_{0}\alpha }_{m{\overline{\mu }}_{E\alpha }}}{h},{ A}_{3}= {\overline{\mu }}_{\rho {C}_{E}},{A}_{4}=\frac{{{\overline{\mu }}_{\gamma }\gamma }_{m}h}{{{\eta }_{0}K}_{m}}, {\omega }_{0}=\frac{{K}_{m}^{*}}{{c}_{0}^{2}{\eta }_{0}{K}_{m}}.$$

Equations (30) and (31) show the system of equations that governs motion and heat transfer and describe transverse oscillations in functionally graded thermoelastic nanobeams. By solving linear partial differential equations, the deviation $w$ and the function $\Theta$, and thus the rest of the areas of the system, can be determined. For this, the Laplace transform method will be used.

## Laplace transform technique

The use of the Laplace transform has proven to be a very effective method for solving linear differential equations with constant coefficients. When using the Laplace transform technique to solve initial value problems, it is usual to use initial conditions, which can be imposed as follows:

34 $$\Theta \left(x,0\right)=\frac{\partial\Theta (x,0)}{\partial t}=0={\text{w}}\left(x,0\right)=\frac{\partial {\text{w}}(x,0)}{\partial t}$$

By utilising the Laplace transform method on the governing Eqs. (30)–(32), we are able to derive the following results:

35 $$\left(\frac{{d}^{4}}{d{x}^{4}}-\frac{\xi {A}_{1}{s}^{2}}{1+{\tau }_{{\text{v}}}^{\alpha }\overline{G }\left(s\right)}\frac{{d}^{2}}{d{x}^{2}}+\frac{{A}_{1}{s}^{2}}{1+{\tau }_{{\text{v}}}^{\alpha }\overline{G }\left(s\right)}\right)\overline{w }=-{A}_{2}\frac{{d}^{2}\overline{\Theta }}{d{x }^{2}},$$

36 $$\frac{{d}^{2}\overline{\Theta }}{d{x }^{2}}=\frac{{s}^{2}\left(1+{\tau }_{0}{\text{s}}\right)}{\left(s+{\omega }_{0}\right)}\left[{A}_{3}\overline{\Theta }-{A}_{4}\left(1+{\tau }_{{\text{v}}}^{\alpha }\overline{G }\left(s\right)\right)\frac{{d}^{2}\overline{w}}{d{x }^{2}}\right],$$

37 $$\overline{M }\left(x,t\right)={A}_{1}\left(\xi {s}^{2}\overline{w }-\left(1+{\tau }_{{\text{v}}}^{\alpha }\overline{G }\left(s\right)\right)\frac{{d}^{2}\overline{w}}{d{x }^{2}}\right)-{A}_{2}\left(1+{\tau }_{{\text{v}}}^{\alpha }\overline{G }\left(s\right)\right)\overline{\Theta },$$

where

38 $$\overline{G}\left( s \right) = \left\{ {\begin{array}{*{20}c} {{\text{s}}^{\alpha } {\text{for}}\;\;{\text{Liouville}} - {\text{Riemann}}\;\;{\text{fractional}}\;\;{\text{derivative}},} \\ {\frac{{{\text{s}}^{\alpha } }}{{{\text{s}}^{\alpha } \left( {1 - \alpha } \right) + \alpha }} {\text{for}}\;\;{\text{Atangana}} - {\text{Baleanu}}\;\;{\text{fractional}}\;\;{\text{derivative}}.{ }} \\ \end{array} } \right.$$

By utilising Eqs. (35) and (36) one can derive the following differential equations that describe the functions $\overline{\Theta }$ and $\overline{w }$

39 $$\left( {D^{6} - {\mathcal{A}}D^{4} + {\mathcal{B}}D^{2} - {\mathcal{C}}{\mathcal{C}}} \right)\left\{ {\overline{\Theta },\overline{w}} \right\}\left( {x,s} \right) = 0,$$

where

40 $$\begin{aligned} {\mathcal{A}} =\, & \delta_{4} + \delta_{1} , {\mathcal{B}} = \delta_{4} \delta_{1} + \delta_{2} + \delta_{5} A_{2} , {\mathcal{C}} = \delta_{4} \delta_{2} ,\delta_{1} = \frac{{\xi A_{1} s^{2} }}{{1 + \tau_{v}^{\alpha } \overline{G}\left( s \right)}}, \\ \delta_{2} =\, & \frac{{A_{1} s^{2} }}{{1 + \tau_{v}^{\alpha } \overline{G}\left( s \right)}},\delta_{3} = \frac{{s^{2} \left( {1 + \tau_{0} s} \right)}}{{\left( {s + \omega_{0} } \right)}},\delta_{4} = A_{3} \delta_{3} ,\delta_{5} = \delta_{3} A_{4} \left( {1 + \tau_{v}^{\alpha } \overline{G}\left( s \right)} \right). \\ \end{aligned}$$

Equation (33) can be rewritten as

41 $$( {D^{2} - {\mathcalligra{h} }_{1}^{2} })( {D^{2} - {\mathcalligra{h}}}_{2}^{2} )( {D^{2} - { \mathcalligra{h}}_{3}^{2} })\{{\bar{\Theta },\bar{w}} \}(x) = 0.$$

The variables ${\mathcalligra{h}}_{n}^{2}$, $n=\mathrm{1,2},3$ in Eq. (41) represent the roots of the equation

42 $${\mathcalligra{h}}^{6}-{\mathcal{A}}{\mathcalligra{h}}^{4}+{\mathcal{B}}{\mathcalligra{h}}^{2}-{\mathcal{C}}=0.$$

The solution to Eq. (41) can be written generally as follows:

43 $${\overline{w },\overline{\Theta }}\left(x,s\right)=\sum_{n=1}^{3}\left\{1,{\beta }_{n}\right\}\left({C}_{n}{{\text{e}}}^{-{\mathcalligra{h}}_{n}x}+{C}_{n+3}{{\text{e}}}^{{\mathcalligra{h}}_{n}x}\right),$$

where $${\beta }_{n}=-\frac{{\delta }_{5}{\mathcalligra{h}}_{n}^{2}}{{\mathcalligra{h}}_{n}^{2}-{\delta }_{4}}$$.

The transformed solution in the Laplace field of the displacement $\overline{u }$ can be derived using Eqs. (16) and (43) as follows:

44 $$\overline{u }\left(x,s\right)=-z\frac{d\overline{w}}{dx }=z\sum_{n=1}^{3}{\mathcalligra{h}}_{n}\left({C}_{n}{{\text{e}}}^{-{\mathcalligra{h}}_{n}x}-{C}_{n+3}{{\text{e}}}^{{\mathcalligra{h}}_{n}x}\right).$$

In order to determine the bending moment $\overline{M }$, the values of the functions $\overline{w }$ and $\overline{\Theta }$ are entered into Eq. (37) in the following manner:

45 $$\overline{M }\left(x,s\right)=\sum_{n=1}^{3}\left({\delta }_{6}-\left({\mathcalligra{h}}_{n}^{2}{\delta }_{7}+{\delta }_{8}{\beta }_{n}\right)\right)\left({C}_{n}{{\text{e}}}^{-{\mathcalligra{h}}_{n}x}{C}_{n+3}{{\text{e}}}^{{\mathcalligra{h}}_{n}x}\right),$$

where

46 $${\delta }_{6}={A}_{1}\xi {s}^{2}, {\delta }_{7}={A}_{1}\left(1+{\tau }_{{\text{v}}}^{\alpha }\overline{G }\left(s\right)\right), {\delta }_{8}={A}_{2}\left(1+{\tau }_{{\text{v}}}^{\alpha }\overline{G }\left(s\right)\right)$$

## Application

When trying to precisely describe the behavior of the nanobeam and forecast how it will react to various loading scenarios, having a solid understanding of the boundary conditions is necessary. These boundary conditions have a substantial impact on the way the nanobeam behaves; specifically, they affect the nanobeam's deformation and the distribution of stress when it is subjected to different loading situations, such as point loads, dispersed loads, or moments.

In the case where both ends of the nanobeam are simply supported, this means that the beam is held in place in a way that allows it to rotate freely and prevents it from moving in any direction perpendicular to its longitudinal axis at the support points. The simply supported boundary conditions for the nanobeam can be expressed as:

47 $$\begin{array}{c}w\left(x,t\right)=0=\frac{{\partial }^{2}w(x,t)}{\partial {x}^{2}}, \mathrm{ at} x=0,\\ w\left(x,t\right)=0=\frac{{\partial }^{2}w(x,t)}{\partial {x}^{2}}, {\text{at}} x=L.\end{array}$$

The heat flux $q\left(x,t\right)$ incorporates thermal effects into the setting of nanobeams, which can affect the nanobeam's temperature distribution, thermal deformation, and structural integrity. In the current work, it is postulated that the initial end of the nanobeam is subjected to a heat flux $q\left(t\right)$ that varies harmonically with time. Therefore, the following thermal conditions are taken into account

48 $$\frac{\partial\Theta }{\partial x}=-q\left(t\right)=-{q}_{0}{\text{cos}}\left(\omega t\right), {\text{on}} x=0.$$

The symbol $\omega$ in Eq. (49) represents the periodical frequency of the flow of heat. The frequency parameter $\omega$ measures the rate at which the heat flow varies with time, providing information on the frequency at which the heat input pattern repeats during a specified period. In a scenario where there is a constant heat flow, $\omega =0$ will be set.

When the thermal insulation is applied to the second end of the nanobeam located at position $x=L$, it signifies the absence of heat transmission at that particular end. This particular boundary condition indicates that the temperature gradient at the terminus of the nanobeam is zero, hence leading to the absence of heat transfer across the boundary. In this case, the following thermal conditions can be taken into account:

49 $$\frac{\partial\Theta }{\partial x}=0 {\text{at}} x=L.$$

When applying the Laplace transform to the boundary conditions (47)–(49), the following equations can be obtained

50 $$\begin{aligned} \overline{w}\left( {x,s} \right) = & 0 = \frac{{d^{2} \overline{w}\left( {x,s} \right)}}{{dx^{2} }}, \quad \quad {\text{ at}} \quad \quad x = 0, \\ \overline{w}\left( {x,s} \right) = & 0 = \frac{{d^{2} \overline{w}\left( {x,s} \right)}}{{dx^{2} }}, \quad \quad {\text{at}}\quad \quad x = L, \\ \end{aligned}$$

51 $$\begin{aligned} \frac{{d{\overline{\Theta }}\left( {x,{\text{s}}} \right)}}{dx} = & - \frac{{q_{0} s}}{{\omega^{2} + s^{2} }} = - G\left( s \right),{ }\quad \quad {\text{at}} \quad \quad x = 0, \\ \frac{{d{\overline{\Theta }}\left( {x,{\text{s}}} \right)}}{dx} = & 0,\quad \quad \quad \quad \quad \quad \quad \quad \quad {\text{at}} \quad \quad x = L. \\ \end{aligned}$$

After inserting Eq. (43) into the previous boundary conditions, the following system of linear equations can be derived:

52 $$\begin{array}{c}\sum_{n=1}^{3}\left({C}_{n}+{C}_{n+1}\right)=0,\\ \sum_{n=1}^{3}\left({C}_{n}{{\text{e}}}^{-{\mathcalligra{h}}_{n}L}+{C}_{n+1}{{\text{e}}}^{{\mathcalligra{h}}_{n}L}\right)=0,\end{array}$$

53 $$\begin{array}{c}\sum_{n=1}^{3}{\mathcalligra{h}}_{n}^{2}\left({C}_{n}+{C}_{n+1}\right)=0,\\ \sum_{n=1}^{3}{\mathcalligra{h}}_{n}^{2}\left({C}_{n}{{\text{e}}}^{-{\mathcalligra{h}}_{n}L}+{C}_{n+1}{{\text{e}}}^{{\mathcalligra{h}}_{n}L}\right)=0,\end{array}$$

54 $$\begin{array}{c}\sum_{n=1}^{3}{\mathcalligra{h}}_{n}\left({{\beta }_{n}C}_{n}-{{\beta }_{n+1}C}_{n+1}\right)=G(s), \\ \sum_{n=1}^{3}{\mathcalligra{h}}_{n}\left({{\beta }_{n}C}_{n}{{\text{e}}}^{-{\mathcalligra{h}}_{n}L}-{{\beta }_{n+1}C}_{n+1}{{\text{e}}}^{{\mathcalligra{h}}_{n}L}\right)=0.\end{array}$$

The indefinite coefficients ${C}_{n}$ ($n=\mathrm{1,2},\dots ,6$) can be found by solving the above system of linear equations. The final step of the solution is to find the inverse Laplace transform.

When applying the Laplace transform method to solve differential equations, the reverse Laplace transform is a critical but challenging phase. Consequently, algorithms for the numerical inverse Laplace transform are frequently employed to compute the outcomes. There have been a great number of numerical inverse Laplace transform methods developed in order to overcome the challenges associated with Laplace transform inversion^68–72^. All the algorithms utilised have produced satisfactory outcomes. Choosing the appropriate algorithm is contingent upon the specific task at hand. Given that each approach has its vulnerabilities, it is advisable to concurrently implement many algorithms in a programme and evaluate the outcomes for comparison.

This work uses the Riemann sum approximation approach together with computer-generated findings to determine the values of altered fields inside the space–time field, employing the Honig and Hirdes algorithm^72^. The Honig and Hirdes methods^72^ are a reliable and quick way to find the numerical inverse Laplace transform, which can be used in the area of fractional-order thermoelasticity. The technique has the potential to be an effective tool for solving differential equations of fractional order.

## Numerical results

In this part, the suggested model will be used to examine how changes in model materials and geometrical parameters affect the thermomechanical vibration properties of FGM beams, considering how these properties change with size. Table 1 presents the mechanical properties of the composite material comprising ceramic and metal components. The nanobeam is constructed exclusively with ceramic material, specifically alumina, on its upper side, located at ${\text{z}}=-h/2$. Conversely, its bottom surface at ${\text{z}}=-h/2$ comprises a metallic material, specifically aluminum.

**Table1:** Values of the mechanical and thermal parameters of the nanobeam.

Characteristics	Unit	Metal (Aluminum (Al))	Ceramic (Alumina (Al_2_O_3_))
Young's modulus ($E$)	${\text{GPa}}$	$70$	$380$
Density ($\rho$)	${\text{kg}}/{{\text{m}}}^{3}$	$2700$	$3800$
Poisson's ratio ($\nu$)		$0.3$	$0.23$
Thermal conductivity ($K$)	${\text{W}}/\left({\text{m}}\text{\hspace{0.17em}}{\text{K}}\right)$	$237$	$1.78$
Thermal diffusivity ($\chi$)	${{\text{m}}}^{2}/{\text{s}}$	$84.18\times {10}^{-6}$	$1.06\times {10}^{-6}$
Thermal expansion (${\alpha }_{t}$)	${{\text{K}}}^{-1}$	$23.1\times {10}^{-6}$	$8.7\times {10}^{-6}$
Reference temperature (${T}_{0}$)	${\text{K}}$	$293$	$293$

**Figure 2:**
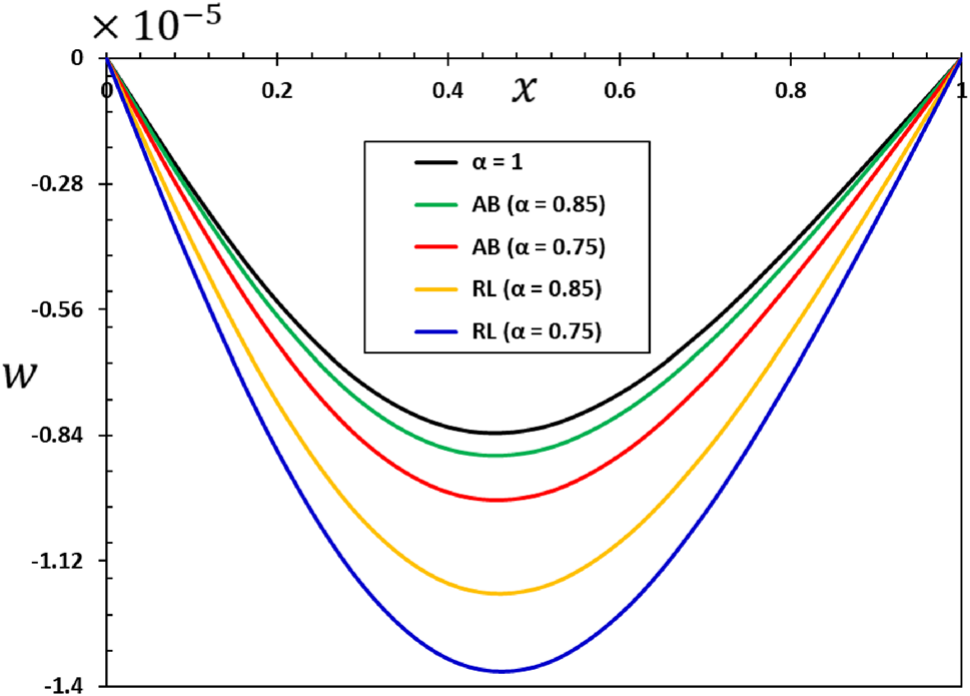
The transverse displacement $w$ against $x$ for different fractional differential operators.

**Figure 3:**
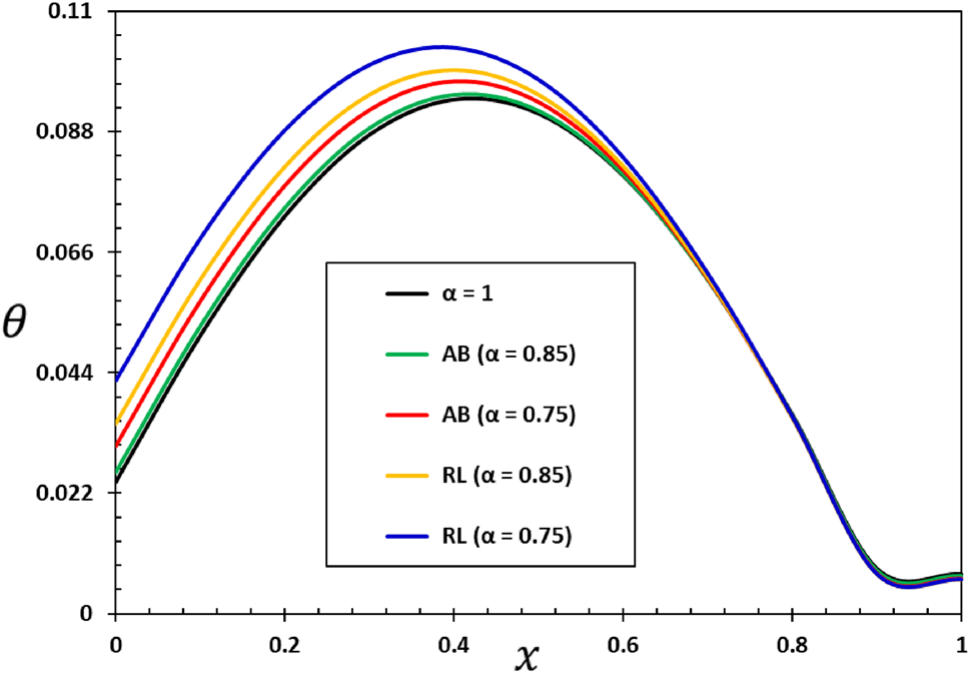
The temperature change $\theta$ against $x$ for different fractional differential operators.

**Figure 4:**
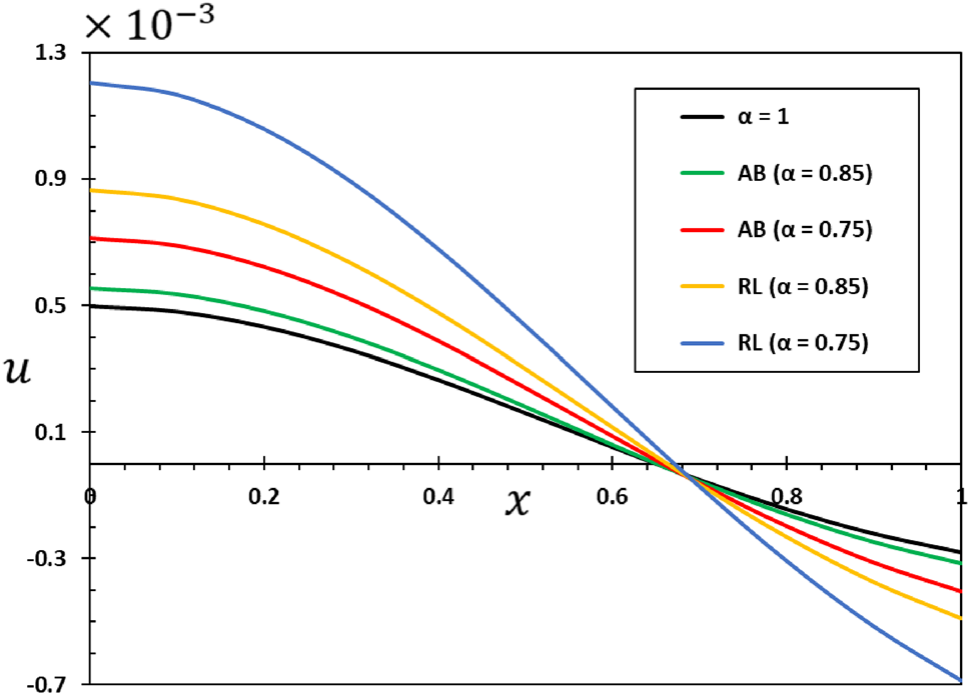
The axial displacement $u$ against $x$ for different fractional differential operators.

**Figure 5:**
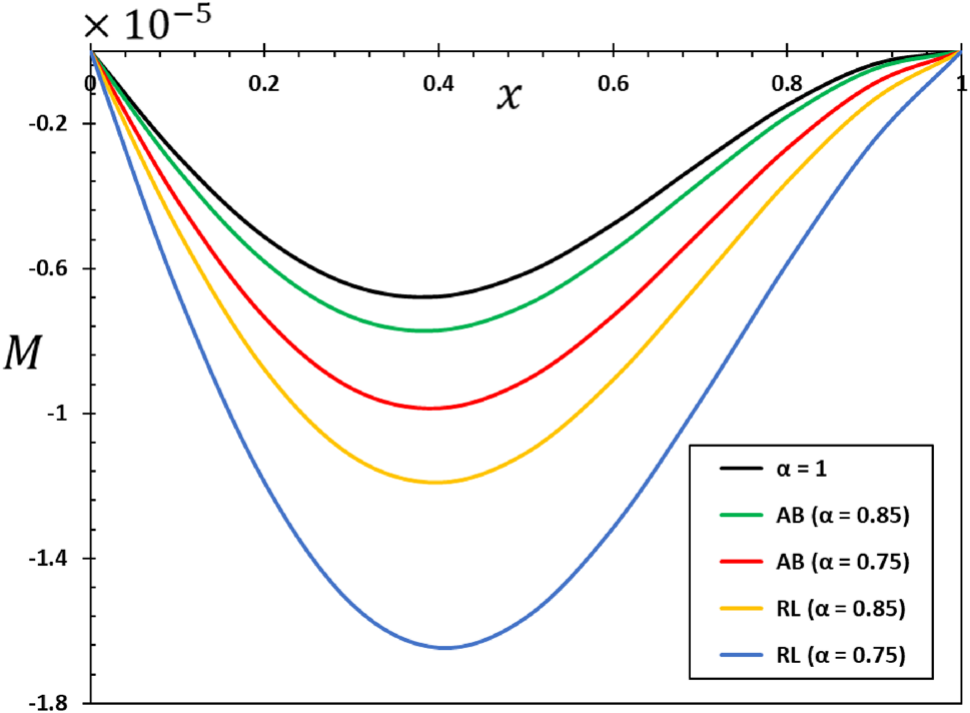
The bending moment $M$ against $x$ for different fractional differential operators.

The ratios $L/h=20$ and $b/h=0.5$ will be chosen based on the assumption that the length of the nanobeam is $L=1\times {10}^{-9}$ metres for the purposes of numerical calculations. In addition, it will be taken into account that the unit of instantaneous time $t$, as well as the phase delay duration referred to as ${\tau }_{0}$, is picoseconds. It has been shown that the value of the nonlocal index ξ of carbon nanotubes typically ranges from 0 to 2.0 nm^73^. Numerical calculations of the non-dimensional physical field variables were performed in the case where the length of the nanobeam changes while the variables $z=h/6$ and $t=0.12$ remain constant. The graphical representations of the numerical computations for the bending moment $M$, temperature change $\theta$, axial displacement $u$, and lateral vibration $w$ are depicted in Figs. 2, 3, 4 and 5. These figures aim to investigate the impact of the viscosity ${\tau }_{{\text{v}}}$, non-local parameter $\xi$ and the fractional-order differential actuators $\alpha$ on the characteristics of these fields. The analysis of numerical computations and graphical representations can be categorized into three distinct case studies.

### Case I: effect of differential operators of fractional order

In order to represent real-world systems characterised by memory effects, the Liouville–Riemann (RL) and Atangana–Baleanu (AB) fractional derivatives offer unique perspectives on fractional derivatives and have distinct advantages and practical applications. Determining which of these alternatives to implement often depends on the specific requirements of the given issue and the formulation's compatibility with the environment of the application. The AB fractional derivative is a variant of the Caputo fractional derivative that integrates the Mittag–Leffler function to model memory impacts accurately. The AB fractional derivative has garnered considerable attention owing to its enhanced capacity to represent memory effects inside certain physical systems correctly.

This section will examine the influence of the AB fractional derivative on the responses of the fields under investigation. The numerical findings and comparisons between variables RL and AB will be visually depicted in graphs 2–5. It is worth noting here that in the case of classical viscoelastic theory with integer derivatives (the conventional Kleven-Voigt model), it is possible to set $\alpha =1$. Otherwise, in the case of modified viscoelastic theory with fractional derivatives (fractional Kleven-Voigt model), $\alpha =0.85$ and $\alpha =0.75$ will be taken into account. In this particular instance, the varying frequency coefficient of the applied heat flow remains fixed at $\omega =5$. In addition, we take $z=h/4$, $\xi =03$, and ${\tau }_{0}=0.02$. The results of this study show that the AB fractional derivative has a big effect on the responses of many domains, especially those with complicated systems that show memory effects. Also, it is important to point out that the Atangana–Baleanu operator has proven to be an indispensable tool for resolving various dynamical systems, including those that the RL operator cannot manage.

Figure 2 illustrates the impact that fractional-order derivatives have on the dimensionless lateral vibration ($w$) behaviour of viscoelastic simply supported nanobeams in the direction of the axial distance $x$. The transverse deflection, $w$, is examined in relation to the distance $x$ while considering various fractional differential operators. Figure 3 shows the effect of fractional actuators on the temperature change $\theta$. Also displayed were the bending moment $M$ and displacement curves $u$ with fractional differential actuators (RL and AB). As shown in Figs. 3 and 4, respectively, it is evident from these figures that modifying the fractional operator, as well as the fractional order parameter, has considerable effects on the behaviour and magnitudes of the various distributions. These changes can be seen in all areas studied. In the same vein, it was discovered that the fractional order coefficient $\alpha$ affects the absolute values of the analysed physical variables, such that a higher value for the fractional order indicator results in higher absolute values. The viscous properties of the nanobeam material may be the main reason for this. It is important to take note of the fact that the deflection $w$ vanishes at the limits of the nanobeam in every scenario; this indicates that it satisfies the boundary requirements that were set for the problem.

When the fractional parameter $\alpha$ changes from $\alpha =0.75$ to $\alpha =1$, it is obvious that the value of the solutions as determined by the fractional derivative operator AB increases. In addition, the results showed that the fractional factor AB has a reducing effect on the magnitudes of field variables and the propagation of mechanical and thermal waves. The figures show that there is a high degree of correspondence between the solutions in the two cases when the values of the order of the fractional derivatives $\alpha$ are close to 1. This indicates that the results obtained from the two forms of rational operators (RL and AB) are consistent when the fractional order approaches the correct order^61,62^.

In addition, when the results of this study are compared to those obtained using the fractional RL derivative, it can be seen that the solutions change very gradually when they are exposed to the fractional AB derivatives. The selection of an appropriate fractional derivative and fractional order is of utmost importance in the modeling process since it can significantly impact the outcomes. Furthermore, it is evident that the procedure for computing the differential equation using the AB derivative is very straightforward and advantageous, a characteristic that is absent in other forms of fractional derivatives^51,52^. The results of this study can be used to learn more about the physical properties of fractional viscoelastic thermoelastic models with fractional differential AB operator.

### Case II: sensitivity to non-local properties

When designing and analysing structures, it is essential to recognise and take into consideration the sensitivity to nonlocal characteristics. Engineers investigate how materials and structures behave under various circumstances and determine how variations in nonlocal characteristics can affect the overall performance using theoretical models, simulations, and experiments. In the field of structural engineering and mechanical sciences, nonlocal factors frequently denote characteristics or circumstances that exert an influence on the performance of a system across a continuum rather than at a discrete location. The consideration of nonlocal characteristics can exert a substantial influence on the general efficiency and reactivity of a structure.

One of the goals of this section is to look into what happens to nano-elastic materials when the non-local theoretical flexible modulus ($\xi$) changes. The rationale behind this phenomenon is the significant influence that this parameter exerts on the mechanical and thermal characteristics of flexible nanobodies. The comprehension and interpretation of these non-local impacts are of the utmost importance in order to accurately describe and construct nanostructures. This is because standard continuum mechanics may not possess the capability to properly comprehend the intricate behaviour observed at these minuscule scales. In order to maintain the dependability and efficiency of elastic nanomaterials and devices, it is imperative for engineers and scientists in the field of nanotechnology to consider and include non-local influences^23,24^.

**Figure 6:**
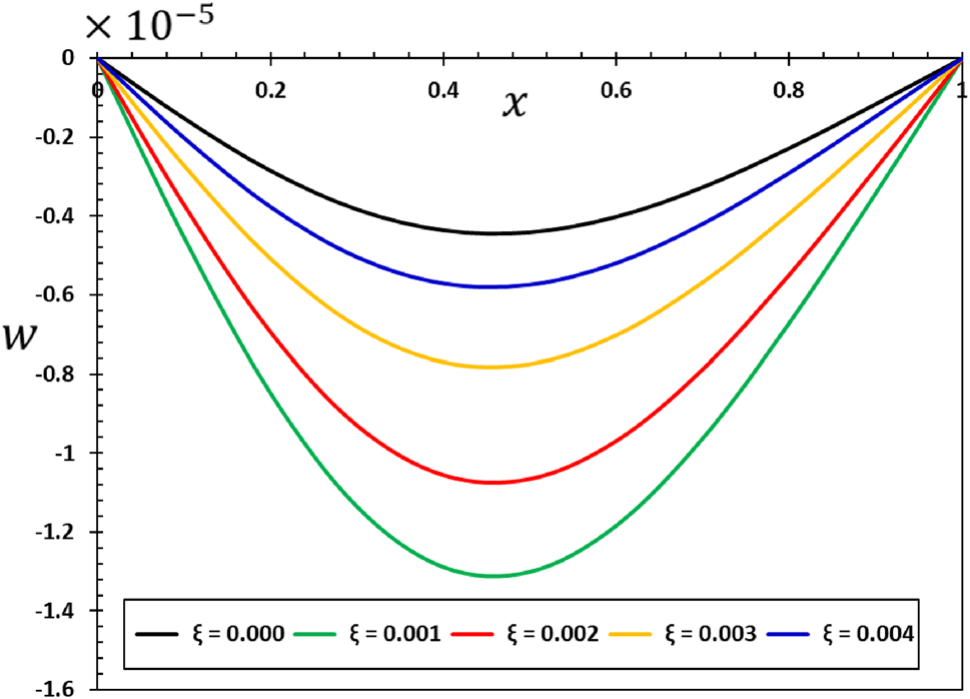
Variation of tangential displacement $w$ versus non-local parameter $\xi$.

**Figure 7:**
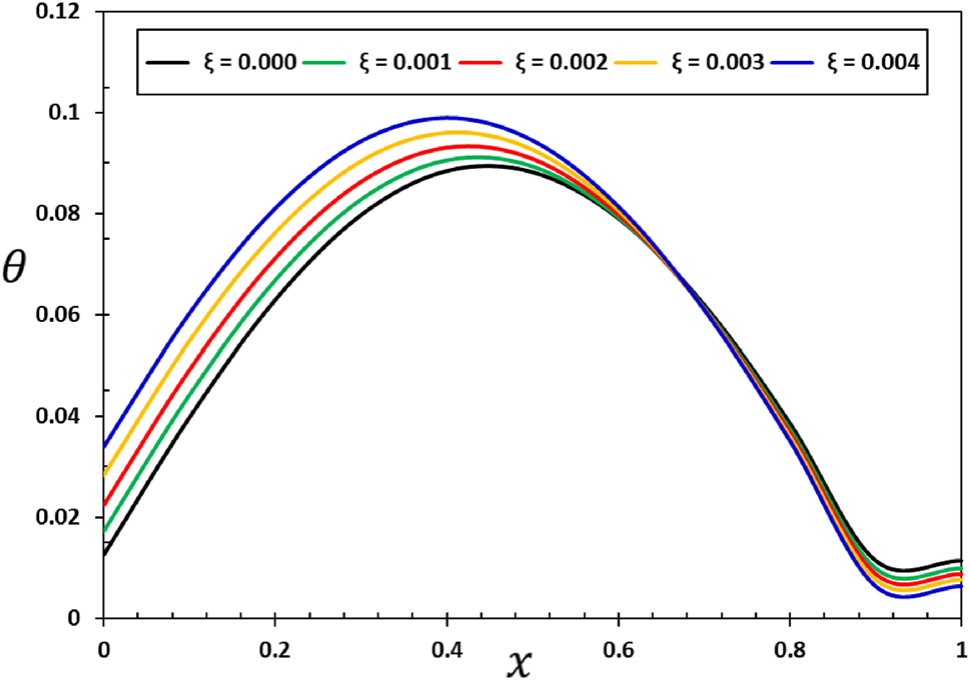
Variation of temperature $\theta$ versus non-local parameter $\xi$.

**Figure 8:**
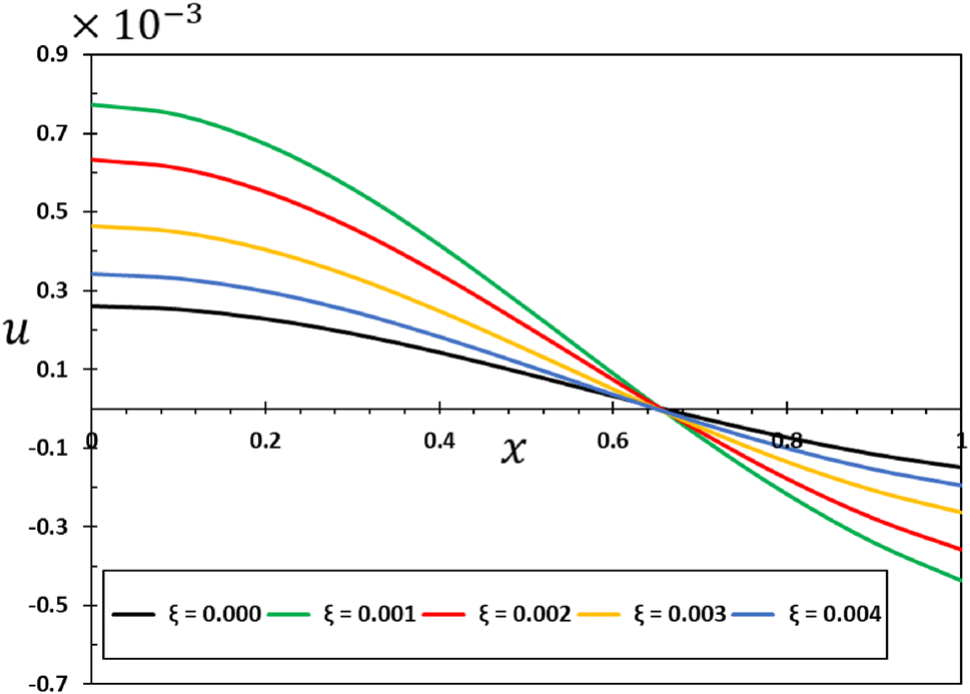
Variation of the axial displacement $u$ versus non-local parameter $\xi$.

**Figure 9:**
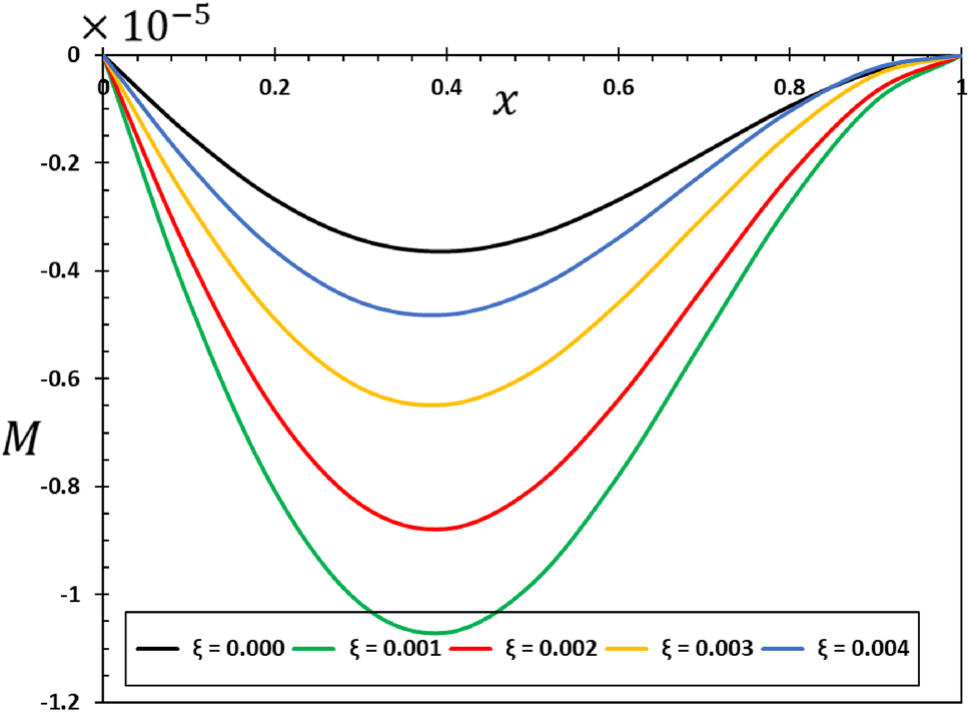
Variation of the bending moment $M$ versus non-local parameter $\xi$.

The influence of the non-local component $\xi$ on the dynamic behaviour of the viscous nanobeams is depicted in Figs. 6, 7, 8 and 9. It is important to highlight that in the given scenario, where $\xi =0$, it corresponds to the conventional case, specifically the local viscoelastic model. In contrast, when considering the values $\xi =0.001$, $\xi =0.002$, $\xi =0.003$, and $\xi =0.004$, it indicates the inclusion of the fractional order non-local viscoelastic theory. In the numerical computations conducted for this particular case study, the periodic frequency parameter of the applied heat flow is held constant ($\omega =3$), as well as other relevant constants such as $\alpha =0.75$, ${\tau }_{0}=0.02$, and ${\tau }_{{\text{v}}}=0.003$. The results shown in Figs. 6, 7, 8 and 9 show that the temperature, deflection, displacement, and bending moment distributions are very sensitive to changes in non-local factors. An increase in the magnitudes of the field variables within the nanobeam has been noticed as the values of the non-local variable $\xi$ grow. Consequently, the results of this study could be useful for future research into many different nanostructure-based systems, such as dampening mechanisms and different ways of designing nanoscale devices^29,74^.

The results in Fig. 6 show that the non-local elastic parameter $\xi$ plays a large role in knowing how the deflection $w$ is distributed in nanoelastic structures. Non-local effects therefore become important at the nanoscale, where the dimensions of the material are very small. This makes the usual assumptions of classical elasticity less valid. Changes in the non-local parameter $\xi$ could cause changes in the way deflection $w$ is distributed, which could affect the stability and integrity of nano-elastic structures.

The data in Fig. 7 shows that non-local processes (non-local elastic parameter $\xi$) can change the way temperature change ($\theta$) in nanoelastic structures. When looking at thermal properties at the nanoscale, there is a lot of complexity. For example, the non-local elastic factor $\xi$ can have a big effect on how heat moves through or leaves a material. Changes in the non-local parameter $\xi$ could cause changes in the temperature profiles, which would have an effect on the thermodynamic properties and performance of nano-elastic systems^18,20^.

The analysis of Fig. 8 shows that the elastic beams and nanostructures can deform in ways different from the conventional case, where the non-local elastic parameter affects the size and shape of the deformation ($u$). Any change or addition to the non-local parameter $\xi$ can cause changes and improvements in the amount of deformation $u$ exhibited by the material, which in turn affects its mechanical response and structural properties. This finding holds great importance since understanding deformation and its regulation at the nanoscale are crucial in the process of creating and reinforcing nanostructures designed for practical purposes. It is also clear from the curves in Fig. 9 that the effect of the non-local theoretical elastic modulus on the bending moment ($M$) variation pattern is essential in the context of elastic nanomaterials and cannot be neglected.

In certain instances, the consideration of nonlocal characteristics can be effectively managed by employing optimisation methodologies, which aim to determine the optimal values of parameters that yield the desired structural performance. This process may entail modifying material qualities, geometric configurations, or other variables in order to attain the necessary equilibrium between strength, stability, and additional performance objectives^22,23^.

### Case III: the effect of viscosity parameter

Nanobeams, characterised by their nanoscale dimensions, possess distinct mechanical properties that deviate from those observed in macroscopic structures. The addition of viscous damping (${\tau }_{{\text{v}}}$) is essential in appropriately describing the dynamic behaviour of nanobeams as they undergo vibrational motion. There is a big effect of the viscous damping coefficient ${\tau }_{{\text{v}}}$, which is also known as the viscosity parameter, on how elastic nanobeams vibrate. Viscous damping (${\tau }_{{\text{v}}}$) is a mechanical phenomenon that converts mechanical energy into heat, resulting in significant impacts on the dynamic behaviour of objects at the nanoscale.

The addition of viscous damping changes the resonance characteristics of nanobeams. The stiffness properties of the material and the characteristics of damping are two important factors that affect the phenomenon of resonance. A thorough examination of damping is important in order to accurately forecast and regulate resonance events in nanobeam constructions. It will be looked into how the viscous damping factor (${\tau }_{{\text{v}}}$) affects the vibrational properties of the Euler–Bernoulli flexible nanobeam in the last scenario of testing and evaluation.

**Figure 10:**
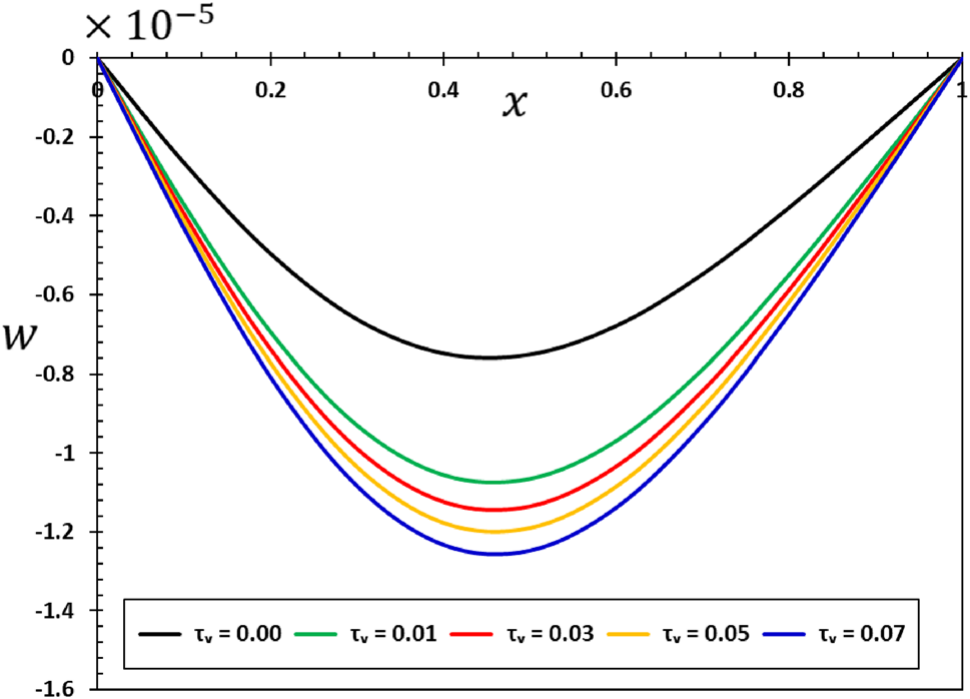
The transverse displacement $w$ versus viscous damping coefficient ${\tau }_{{\text{v}}}$.

**Figure 11:**
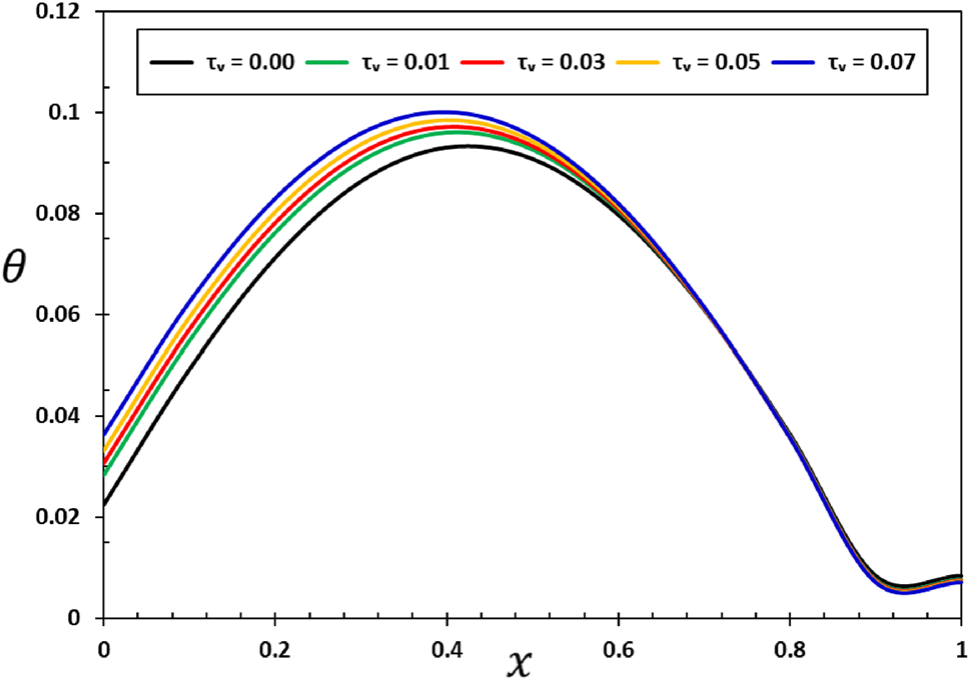
The temperature change $\theta$ versus viscous damping coefficient ${\tau }_{{\text{v}}}$.

**Figure 12:**
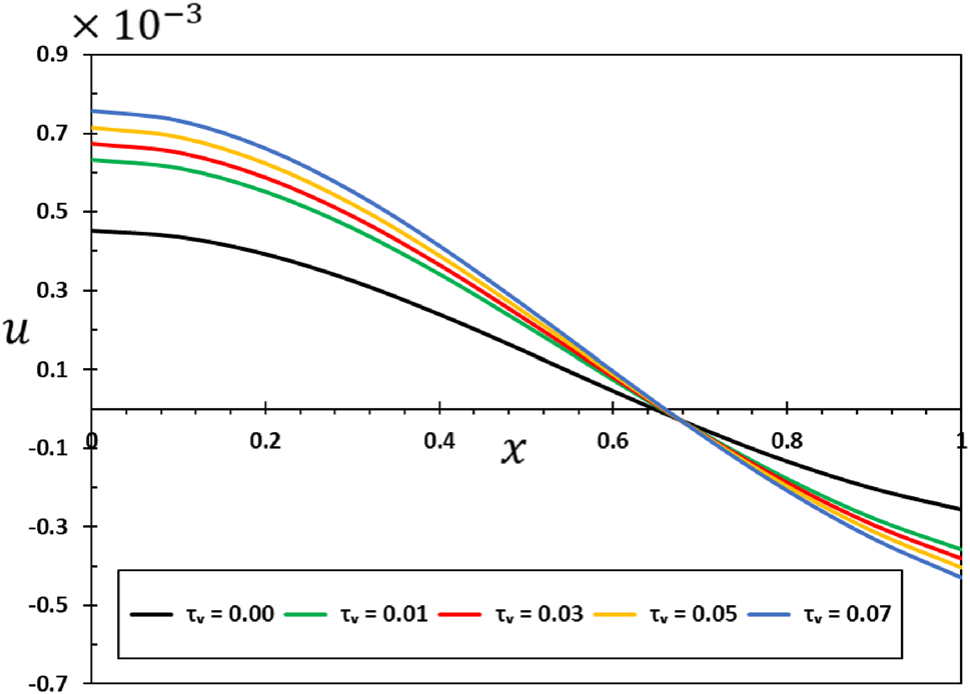
The axial displacement $u$ versus viscous damping coefficient ${\tau }_{{\text{v}}}$.

**Figure 13:**
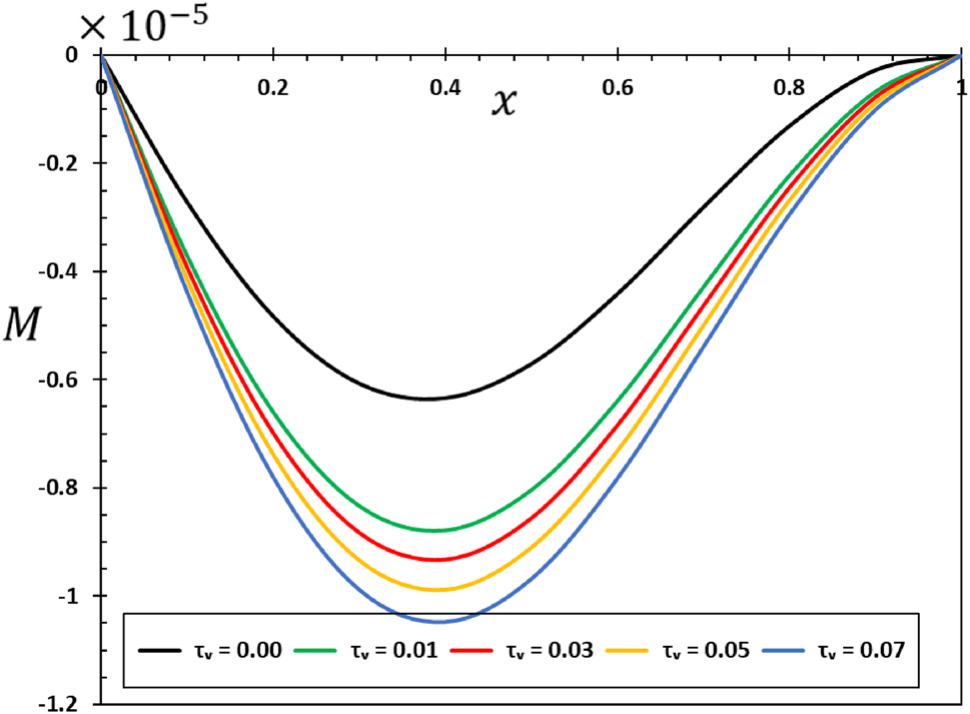
The bending moment $M$ versus viscous damping coefficient ${\tau }_{d}$.

It is noteworthy that in the case of an elastic material, where the viscosity parameter (${\tau }_{{\text{v}}}$) is equal to zero, the outcomes of the elastic nonlocality concept are demonstrated. In the numerical calculations in this case, it was taken into account that the other effective constants are constant ($\alpha =0.75$, $\xi =0.003$, ${\tau }_{0}=0.02$, and $\omega =3$). Figures 10, 11, 12 and 13 show the effect of the viscous damping coefficient and viscosity on the deflection, deformation, temperature change, and bending moment behaviour of nanobeams due to viscous damping ${\tau }_{{\text{v}}}$. The figures demonstrate that viscous damping plays a significant role in facilitating energy dissipation mechanisms within the system. Furthermore, its influence extends to multiple facets of the dynamic and thermal behavior shown by the nanobeam.

It is evident from Figs. 10 and 12 that the viscous damping coefficient has a considerable impact on the deflection and deformation patterns of the viscous nanobeam. Greater damping coefficients lead to a quicker loss of vibrational energy, which in turn leads to a greater amplitude of deflection and distortion^47,50^. This is of utmost significance in nanomechanical systems, as accurate control of deflection is essential for their operation^56^.

Figure 13 clearly shows how the viscous damping (${\tau }_{{\text{v}}}$) changes the way bending moments are distributed in the nanobeam when it is dynamically loaded with harmonic heat flux. As the level of damping increases, both the amplitude and duration of the bending moment can decrease. The comprehension of the structural integrity of nanoscale systems under dynamic forces is of utmost importance.

The dynamic stability of the nanobeam is significantly influenced by the viscosity and damping parameter ${\tau }_{{\text{v}}}$. The relationship between stiffness, damping, and viscosity impacts whether the amount of vibration remains limited or expands exponentially over time. Comprehending its inherent stability is crucial in order to forecast the extended-term performance of the nanobeam. In conclusion, a crucial factor in understanding how elastic nanobeams vibrate is the viscous damping coefficient^59,60^. For the purpose of designing and optimising nanomechanical systems and devices, it is imperative that their incorporation into mathematical models be used in order to precisely forecast and comprehend the changing behaviour of viscoelastic nanostructures^61^.

## Conclusion

The current study presents the incorporation of the Kelvin–Voigt fractional viscoelastic model into the FG Euler–Bernoulli nanobeam, utilising the nonlocal elastic theory and the Atangana–Baleanu (AB) fractional derivative. This approach aims to characterise the dynamic response of nanostructures, encompassing viscoelastic properties and the influence of small-scale phenomena. We use this model to study and develop functionally graded nanomechanical systems because it is crucial to accurately predict their viscoelastic response. We assume that the characteristics of the nanobeam gradually vary across its thickness, transitioning from a purely ceramic composition to a purely metallic composition. This study examines and analyses the impact of many factors, including fractional order, damping parameters, and nonlocality, on the vibrational characteristics of nanobeams. Based on the results of this research, the following conclusions can be emphasised:

- By adding fractional derivatives to the Kelvin–Voigt model in the context of fractional derivative viscoelasticity, we can represent the memory and heredity effects in the material's response.
- The Kelvin–Voigt fractional derivative viscoelastic model describes how the nanobeam moves and reacts to outside forces or moments. It does this by showing how the beam bends and deforms.
- The results showed that the fractional factor AB has a reducing effect on the magnitudes of field variables and the propagation of mechanical and thermal waves.
- Changes in the non-local parameter could alter the distribution of deflection, which could impact the stability and integrity of nano-elastic structures.
- Any change or increase in non-local parameter values can lead to changes and enhancements in the material's deformation, subsequently influencing its mechanical response and structural properties. This finding holds great importance since understanding deformation and its regulation at the nanoscale are crucial in the process of creating and reinforcing nanostructures designed for specific purposes.
- Higher viscosity materials are likely to experience more dramatic temperature changes when viscous nanobeams undergo thermal loading. The change in temperature also has a direct effect on the damping process, which in turn can affect the overall thermal stability of the nanobeam.

Finally, critical factors in understanding how viscoelastic nanobeams vibrate are the viscous damping coefficient and nonlocality. To design and improve nanomechanical systems and devices, we must use mathematical models to accurately predict and understand how viscoelastic nanostructures change over time.

## Data Availability

All data generated or analysed during this study are included in this published article [and its supplementary information files].
